# Polypus of the Rectum

**Published:** 1847-04

**Authors:** 


*Polypus of the Rectum.* By Mr. Syme.—Sir A. Cooper states, in his Surgical Lectures, that "in the course of his life," he met with only ten cases of polypus of the rectum.

Some time ago, I met with five cases in the course of a single fortnight—two of them in adults, and three in children—and I have seen a sufficient number of other instances of the disease, to satisfy me that it is not by any means so rare as has generally been supposed. It presents itself in three different forms, of which one usually occurs in childhood, and does not appear much beyond puberty. A gentleman now established in practice, not far from Edinburg, when attending my lectures—then I suppose about eighteen or nineteen years of age—applied to me for the removal of a polypus, such as is met with in early life; with this exception, I never met with it beyond the ninth or tenth year. It is extremely soft vascular, of a florid red colour, and assumes the form either of a worm from two to four inches in length, or of a strawberry with a connecting footstalk two or three inches long. This tumor seldom protrudes except when the bowels are evacuated, and then admits of ready replacement, although not without occasional hæmorrhage, which may be of considerable amount. The vascularity of this growth, and its attachment above the sphincter, made me averse from removing it by excision; and Sir A. Cooper has mentioned the alarm that was on one occasion excited in his practice by doing so. I have always employed the ligature; and though the soft structure readily gives way when the thread is drawn, bleeding has never occurred in a single instance, or any other symptom in the least degree disagreeable resulting from this mode of removal; I am therefore induced to regard it as the best that can be employed.

The disease appears in adults in two very distinct forms. In one of these, the growth is soft, vascular, prone to bleed, lobulated or shreddy, and malignant-looking, so as on the whole to resemble very much the cauliflower excrescence of the os uteri, but possesses a peduncle or foot stalk of firm texture, capable of sound cicatrization after being divided. The profuse, frequent, and protracted bleeding which proceeds from this sort of growth, renders its removal an object of great consequence; and this may be effected very easily, and with perfect-safety, by transfixing the radical cord of connexion with a double ligature, tying the threads so as to include a half of it in each, and then cutting it across a little below the constricted part. In a patient of Mr. Craig of Ratho,(who detected the disease from the great hæmorrhage it occasioned,) I could not accomplish protusion of the tumor, but guided a ligature on my finger, and tied it on the neck within the rectum. It is more satisfactory to force or draw the swelling beyond the sphincter, so that the sound and morbid parts may be distinguished with certainty, and this can usually be done with great facility, although the growth has attained a large size. In a hospital case recommended by Mr. Anderson of Castle-Douglass, I brought into view and removed a tumor not less than an orange, which had a most malignant aspect, and had nearly exhausted the patient by hæmorrhage.

In the other form which polypus of the rectum assumes in adults, the tumor is of a firmer consistence, smoother surface, and more regularly spherical or oval form, so as to resemble the growth which in general constitutes *polypus uteri.* The symptoms resulting from this simple swelling are rather annoying than seriously alarming; and the patient, therefore, is apt to delay requiring assistance for a long while. In the case of an old lady, whom I saw with Mr. Hilson of Jedburgh, the tumor was about the size of a cherry, with a long stalk, and we were assured had protruded every time the bowels moved for twenty years. In another case, a gentleman whom I saw with Dr. Johnston of Cumnock, the tumor was nearly as large as an egg, had a cuticular covering, and appeared to have existed for a period equally long. I have always removed these growths in the way that has been already described, and never met with the slightest consequence of a disagreeable kind.—*Lond. and Ed. Month. Jour. of Med. Science.*

